# Wnt gene expression in human trabecular meshwork cells

**Published:** 2010-01-28

**Authors:** Rajalekshmy Shyam, Xiang Shen, Beatrice Y.J.T. Yue, Kelly K. Wentz-Hunter

**Affiliations:** 1 Department of Ophthalmology and Visual Sciences, University of Illinois at Chicago College of Medicine, Chicago, IL; 2 Roosevelt University, Department of Biological, Chemical and Physical Sciences, Chicago, IL

## Abstract

**Purpose:**

The aim of this study was to examine the expression of genes related to the Wnt signaling pathway, such as β-catenin (*CTNNB1*) and secreted frizzled-related protein-1 (*sFRP1*), in human trabecular meshwork (TM) cells. In addition, the effect of oxidative stress on Wnt signaling was evaluated.

**Methods:**

All experiments were conducted using second- or third-passaged human TM cells. cDNA was prepared from total RNA extracted from cells by means of reverse transcription. PCR was then performed to determine the presence of Wnt genes. For oxidative stress, TM cells were treated with 1 mM of H_2_O_2_ for 30 min. Actin staining was carried out to verify cell response to oxidative stress. Western blotting was used to measure Wnt-related protein levels after H_2_O_2_ treatment.

**Results:**

Positive PCR products were detected for a total of 25 Wnt and Wnt-related genes in human TM cells. Most of the genes identified belonged to the Wnt/β-catenin pathway. Members of the β-catenin-independent noncanonical pathways were also found. Oxidative stress did not result in significant changes in β-catenin and sFRP1 protein levels.

**Conclusions:**

Genes related to canonical and noncanonical Wnt pathways are expressed in human TM cells. It appears that all three Wnt pathways are operative in the TM system. Oxidative stress, while thought to play a role in the development of glaucoma, had little effect on the Wnt activity in TM cells.

## Introduction

The trabecular meshwork (TM) located at the chamber angle of the eye is the major site for regulation of the aqueous humor outflow [1]. Cells in the TM are believed to play an essential role in maintenance of the outflow system and control of intraocular pressure (IOP). Dysfunction or alteration of TM cell activities may be responsible for the development of glaucoma, a disease frequently associated with IOP elevation and neural and visual loss.

The Wnt gene family encodes secreted glycoproteins that are highly conserved across a variety of species [2]. These proteins have been shown to be involved in diverse biologic processes, including embryonic induction, generation of cell polarity, and the specification of cell fate [3]. Many Wnt genes have also been implicated in proto-oncogenic activities [4].

The Wnt genes signal through a β-catenin-dependent canonical pathway as well as two β-catenin-independent noncanonical pathways. Central to the canonical pathway is the regulation of β-catenin activity. In the absence of Wnt ligands, β-catenin accumulated in the cytoplasm is phosphorylated by glycogen synthase kinase3β (GSK3β) in a multiprotein destruction complex composed of proteins, including axin and the adenomatous polyposis coli (APC) tumor suppressor protein, and is thereby marked for degradation through the ubiquitin–proteasome system. When the Wnt ligand binds to membrane receptor frizzled (Fzd) and the low-density lipoprotein receptor-related protein 5/6 (LRP5/6), the cytoplasmic target of Fzd, disheveled (Dsh), is activated to suppress the GSK3β activity. As a result, β-catenin is neither phosphorylated nor degraded, and the excess β-catenin is translocated into the nucleus [5]. Once there, β-catenin associates with the T-cell factor (TCF)/lymphoid enhancer factor (Lef) transcription factors to induce specific gene expression [6]. In the noncanonical planar cell polarity (PCP) pathway, Dsh is connected to downstream effectors, such as small GTPase Rho, to regulate cytoskeletal organization and cell polarity. The second noncanonical pathway Wnt/Ca^2+^ leads to release of intracellular Ca^2+^ and involves activation of protein kinase C and Ca^2+^/calmodulin-dependent protein kinase II. The Wnt/Ca^2+^ pathway has implications on cell proliferation and cell movement [7].

Many extracellular inhibitors of Wnt signaling have been reported. One example is the secreted frizzled-related protein (sFRP), which antagonizes Wnt signaling by sequestering the Wnt protein in the extracellular matrix [8]. Another inhibitor, Dickkopf (Dkk), antagonizes Wnt through interactions with LRP5/6 [9], although it can also act as an activator of the noncanonical Wnt/PCP pathway [10].

A possible role of Wnt signaling in the outflow system has been suggested recently [11]. An increased expression of sFRP1 appeared to result in IOP elevation in mice [11]. In search of Wnt and Wnt-related genes, six of them were identified in the TM [11]. To extend these previous efforts, we comprehensively examined the expression patterns of genes in Wnt signaling pathways in human TM cells. Additionally, we undertook an investigation to determine whether Wnt signaling was altered by oxidative stress in TM cells. Oxidative stress has long been thought to be involved in the development of glaucoma [12-16]. Regulation of Wnt activity by oxidative stress has previously been reported in several other cell types [17-19].

## Methods

### Cell culture

TM tissues were dissected from corneo-scleral rims obtained from donors 32, 33, 43, 51, and 58 years of age (Illinois Eye Bank, Chicago, IL). The tissues were plated onto Falcon Primaria flasks (Becton-Dickinson Co., Oxnard, CA). At least 2 ml of complete medium [20,21] containing Eagle’s minimum essential medium (Sigma, St. Louis, MO), 5% calf serum (Sigma), 10% fetal bovine serum (Sigma), essential (Invitrogen, Carlsbad, CA) and nonessential (Sigma) amino acids, and antibiotics (Sigma) was added to each flask. Cells were trypsinized and subcultured at confluence. Second- or third-passaged cells were used in all the experiments. All experiments were conducted when cells were at confluence for at least 2 days.

### Hydrogen peroxide treatment

To study the effects of oxidative stress, cells at confluence for at least 2 days were treated with 1 mM hydrogen peroxide (H_2_O_2_; Sigma) in serum-free complete medium (Sigma) for 30 min [22]. Total RNA and cell lysates were collected at the subsequent 0-, 1-, 2-, or 4-h time points. Untreated cells were used as controls.

### cDNA synthesis and PCR

Total RNA was isolated from cultured TM cells, using the RNeasy mini kit (Qiagen, Valenica, CA). The RNA concentration was measured using an ND-1000 spectrophotometer (NanoDrop Technologies, Wilmington, DE). At least 1 μg of RNA was used for cDNA synthesis, employing random hexamers and the Superscript RT first strand synthesis kit (Invitrogen). PCR was conducted on aliquots of cDNA samples by using Multigene thermocycler (Labnet International, Woodbridge, NJ). Each reaction mixture contained 5–10 μM of gene-specific primer pairs, 0.5–1 μg of cDNA, and PCR master mix containing 1.5 mM MgCl_2_ and 200 µM of dNTPs (Promega, Madison, WI) in a volume of 50 μl. The primers, designed using the OligoPerfect™ Designer (Invitrogen) program, were purchased from either Sigma or IDT (Coralville, IA). The sequences of primer pairs for target genes along with the annealing temperatures and expected product sizes are listed in Table 1.

**Table 1:** Primer pair sequences, annealing temperature, and product sizes of Wnt-related genes.

**Genes examined**	**Forward primer**	**Reverse primer**	**Annealing temperature (°C)**	**Expected product size (bp)**
*Wnt1*	5’-TCCTCCACGAACCTGCTTAC-3’	5’-GCCTCGTTGTTGTGAAGGTT-3’	54	491
*Wnt2*	5’-GGTGATGTGCGATAATGTGC-3’	5’-GCCAGCTCTGTTGTTGTGAA-3’	52	404
*Wnt2b*	5’-AAGATGGTGCCAACTTCACC-3’	5’-GGCCACAGCACATGATTTCAC-3’	54	188
*Wnt3*	5’-CTGTGAGGTGAAGACCTGCTG-3’	5’-GATGCAGTGGCATTTTTCCT-3’	54	357
*Wnt3a*	5’-GGTGGCTGTAGCGAGGACAT-3’	5’-ATGCCGTGCGAGCTGACGTT-3’	56	454
*Wnt4*	5’-GCATCTCAGAGGAGGAGACG-3’	5’-TCAGAGCATCCTGACCACTG-3’	56	363
*Wnt5a*	5’-ATTTTTCTCCTTCGCCCAGGT-3’	5’-GGCTCATGGCGTTCACCAC-3’	55	358
*Wnt5b*	5’-CCAAAGGATCAGAGGAGCAG-3’	5’-TACACCTGACGAAGCAGCAC-3’	56	483
*Wnt6*	5’-TGGTTATGGACCCTACCAGCA-3’	5’-CGTCCATAAAGAGCCTCGAC-3’	54	467
*Wnt7a*	5’-CTGGAGGAGAACATGAAGC-3’	5’-ACAGCACATGAGGTCACAGC-3’	54	354
*Wnt8a*	5’-TCCCAAGGCCTATCTGACCTAC-3’	5’-CCGGCCCTGTTGTTGTGA-3’	57	407
*Wnt10b*	5’-AATGCGAATCCACAACAACA-3’	5’-ACAGCACATAGCAGCACCAG-3’	54	448
*Wnt11*	5’-CTACACAACAGTGAAGTG-3’	5’-CCCACCTTCTCATTCTTCATGC-3’	55	298
*Dsh1*	5’-CACCCTGAACCTCAACAGTGG-3’	5’-CCCTTCACTCTGCTGACTCC-3’	56	201
*Dsh2*	5’-CCTTCAGCAGCGTCACAGATTCC-3’	5’-AGTGGGCAGCAGGGGCC-3’	59	895
*Dsh3*	5’-CAGCCCCCTTCTGTGCTGATAA-3’	5’-AGAAGGTGATCTTGTTGA-3’	59	1196
*Fzd1*	5’-TGCGAGGCGCTCATGAACAA-3’	5’-CCTCGGCGAACTTGTCATTA-3’	54	570
*Fzd2*	5’-CGTCCTCAAGGTGCCATCCTA-3’	5’-CAGCCCGACAGAAAAATGAT-3’	54	248
*Fzd4*	5’-CTCGGCTACAACGTGACCAAGAT-3’	5’-AATATGATGGGGCGCTCAGGGTA-3’	57	604
*Fzd5*	5’-CTGCTACCAGCCGTCCTTCAGT-3’	5’-CCATGCCGAAGAAGTAGACCA-3’	59	319
*Fzd7*	5’-CGACGCTCTTTACCGTTCTC-3’	5’-GCCATGCCGAAGAAGTAGAG-3'	54	246
*Fzd8*	5’-GGACTACAACCGCACCGACCT-3’	5’-ACCACAGGCCGATCCAGAAGAC-3’	59	406
*LRP5*	5’-AAGATCATTGTGGACTCGGAC-3’	5’-GAAAGGCTCGCTTGGG-3’	52	395
*LRP6*	5’-ACTGTATCCCTGTGGCTTGG-3’	5’-CCCTTCATACGTGGACACA-3’	54	426
*CTNNB1*	5’-CATGGAACCAGACAGAAAAGC-3’	5’-GCTACTTGTTCTTGAGTGAAG-3’	52	200
*GSK3β*	5’-GCAGCAGCCTTCAGCTTTTGG-3’	5’-CCGGAACATAGTCCAGCACCAG-3’	59	358
*APC*	5’-TCCACAACATCATTCACTCACAG-3’	5’-TGCTCGCCAAGACAAATTCC-3’	53	505
*TCF1*	5’-GAGCAAAGAGGCACTGATCC-3’	5’-CTGGTTGAGGCCAGTGGTAT-3’	54	361
*TCF3*	5’-GGGTCTTCCATCCTCGGTGTA-3’	5’-GAGTAGATCGAGGCCAGTGC-3’	56	488
*TCF4*	5’-GTTTGTATTTTTTGGCG-3’	5’-GAATGGCTGCCTTAGGG-3’	49	467
*TCF7*	5’-GAGCCAAGGTCATTGCAGAGT-3’	5’-GTGGTGGATTCTTGGTGCTT-3’	54	222
*Dkk1*	5’-GATCTGTAAACCTGTCCT-3’	5’-GAAGAATTACTGGCTTGATG-3’	50	149
*Dkk2*	5’-CTGATGGTGGAGAGCTCACAG-3’	5’-ATTATTGCAGCGGGTACTGG-3’	56	311
*sFRP1*	5’-CAACCTGCTGGAGCACGAGAC-3’	5’-CGCTGGCACAGAGATGTTCA-3’	58	379
*sFRP2*	5’-CACGGCATCGAATACCAGAAC-3’	5’-GATGCAAAGGTCGTTGTCCT-3’	58	308
*sFRP3*	5’-GGGCTGTGAGCCCATACTCAT-3’	5’-GGCAGCCAGAGCTGGTATAG-3’	57	379

Thermocycling conditions were 94 °C for 5 min, followed by 35 cycles of 94 °C for 30 s, primer-specific annealing temperature for 30 s, 72 °C for 30 s, and a cycle of 72 °C for 10 min. Negative controls with RNA samples that were not subjected to reverse transcription were included for all PCR experiments. PCR products, along with Marker VI (Roche Laboratories, Nutley, NJ) or GeneRuler^TM^ 100-bp ladder markers (Fermentas, Hanover, MD) were resolved on 1% agarose gels. The gel images were captured with the Gel Doc 2000 image analyzer (Bio-Rad, Hercules, CA). Sequencing analyses were performed to verify the identities of all PCR products. At least two independent experiments, using cells from two different donors, were performed.

A human breast adenocarcinoma cell line, MCF-7, is known to express *Wnt2*, *Wnt2b*, *Wnt3*, *Wnt3a*, *Wnt4*, *Wnt10b*, *Wnt11*, *sFRP1*, *sFRP2*, β-catenin (*CTNNB1*), and *LRP5* [23-29]. MCF-7 cDNAs were used as positive control for these genes. Human embryonic kidney 293 (HEK293) cells were used as a positive control for *TCF1* gene expression as this transcription factor has been shown to be expressed in the kidney [30].

### Western blotting

Lysate was prepared from TM cells after H_2_O_2_ treatment, using the CelLytic M reagent (Sigma) in the presence of protease inhibitors (Roche). Total protein was quantified by the bicinchoninic acid assay (Thermo Scientific, Rockford, IL). At least 10 µl of cell lysate was used to determine the protein content. Bovine serum albumin was used as a standard. Protein concentration was determined using Tecan GeniosPro microplate reader (Tecan, Research Triangle, NC). Equal amount of protein from each sample resolved on a 10% sodium dodecyl sulfate (SDS)-polyacrylamide gel under reducing conditions was electroblotted onto a Protran nitrocellulose membrane (Midwest Scientific, St. Louis, MO). The membrane was blocked with 5% nonfat dry milk and incubated with monoclonal anti-β-catenin (1:1,000; Santa Cruz Biotechnology, Inc., Santa Cruz, CA) or polyclonal anti-sFRP1 (1:1,000; Abcam, Cambridge, MA). Horseradish peroxidase-conjugated anti-rabbit or anti-mouse IgG (1:10,000; Jackson ImmunoResearch Laboratories, West Grove, PA) was used as the secondary antibody. Protein bands were detected using HyGLO Chemiluminescent HRP antibody detection kit (Denville Scientific, Metuchen, NJ). The blot was also stripped using ImmunoPure IgG Elution buffer (Thermo Scientific) for 30 min at room temperature and reprobed with polyclonal anti-glyceraldehyde 3-phosphate dehydrogenase (GAPDH; 1:5,000; Trevigen, Gaithersburg, MD) for protein loading control. Densitometry was performed and Wnt protein levels were normalized against that of GAPDH. At least three independent experiments were conducted. Statistical analyses were performed using the Student’s t test.

### Actin staining

After a 30-min treatment with 1 mM H_2_O_2_, TM cells in chamber slides were fixed immediately after (0 h) or 4 h later with fixative containing 2% paraformaldehyde, 0.08 M lysine, and 10 mM sodium periodate [22] for 20 min. The cells were permeabilized in 0.1 M sodium phosphate buffer containing 0.2% Triton X-100, 0.1% bovine serum albumin for 8 min, and were allowed to react at room temperature with Alexa Fluor 488 phalloidin (1:30; Invitrogen) for 30 min. They were then mounted in Vectashield (Vector Laboratories, Burlingame, CA) and photographed, using Axioscope (Carl Zeiss MicroImaging, Thornwood, NY).

## Results

**Figure 1:**
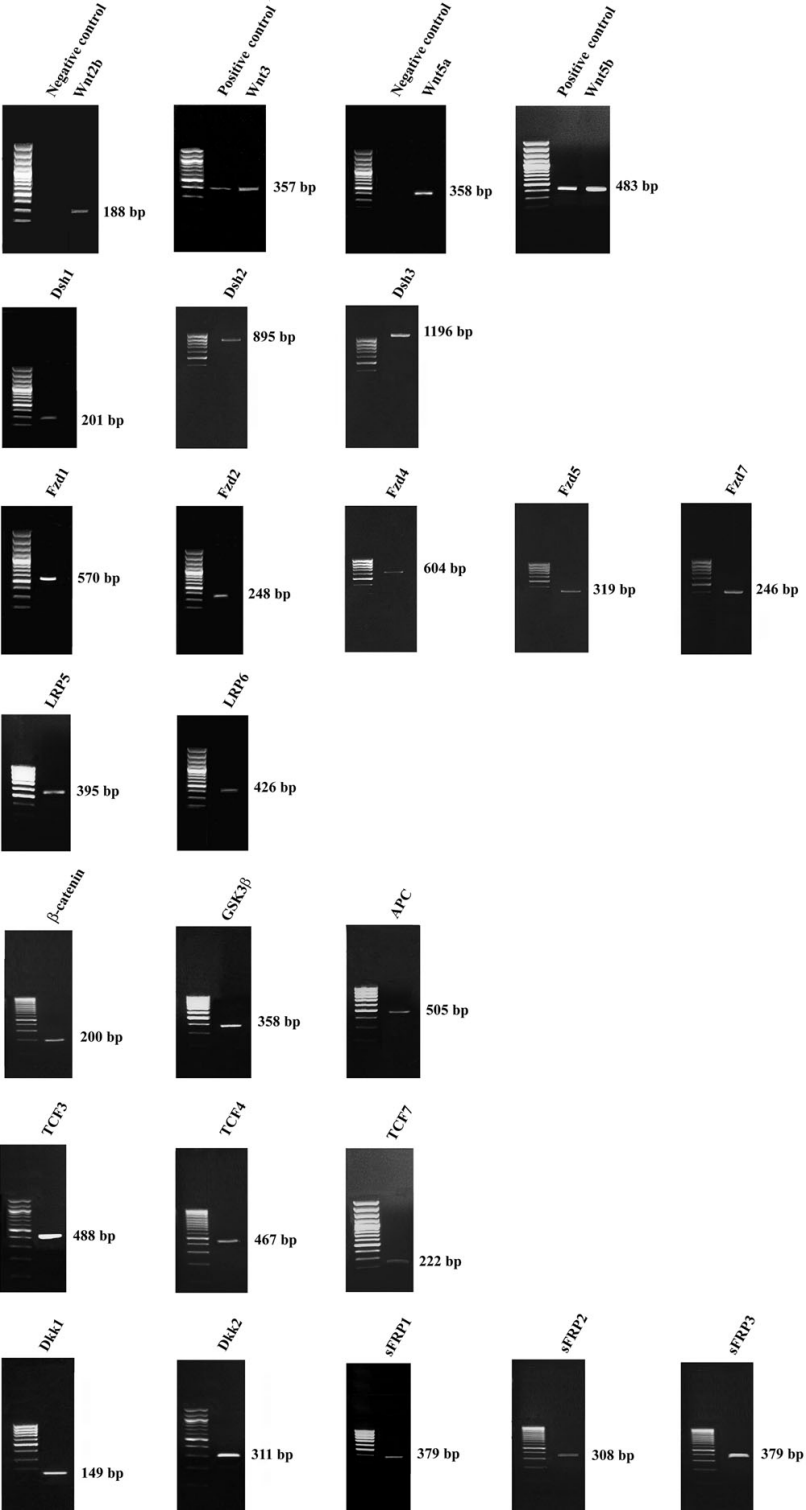
Expression profile of Wnt ligands and Wnt-related genes in human trabecular meshwork cells. Total RNA extracted from cells was reverse transcribed and amplified with specific primers for Wnt-related genes listed in Table 1. PCR products were resolved on a 1% agarose gel and visualized by ethidium bromide staining. Positive PCR products were obtained for *Wnt2b*, *Wnt3*, *Wnt5a*, *Wnt5b*, *Dsh1*, *Dsh2*, *Dsh3*, *Fzd1*, *Fzd2*, *Fzd4*, *Fzd5*, *Fzd7*, *LRP5*, *LRP6*, *CTNNB1*, *GSK3β*, *APC*, *TCF3*, *TCF4*, *TCF7*, *Dkk1*, *Dkk2*, *sFRP1*, *sFRP2*, and *sFRP3*. Negative controls with RNA samples that were not subjected to reverse transcription were included for all PCR reactions. No PCR products were seen with any of the negative controls (representative results are shown for *Wnt2b* and *Wnt5a*). When applicable, positive controls, using MCF-7 (shown for *Wnt3* and *Wnt5b*) and HEK293 cDNAs, were performed in parallel. All PCR products were confirmed by sequence analyses, and all the experiments were performed in at least two different cell lines from two different donors.

The expression in human TM cells of a total of 36 genes (Table 2) in the Wnt signaling pathway that included 13 Wnt ligands, three transduction (Dsh) genes, eight receptors, *CTNNB1*, *GSK3β*, *APC*, four TCF transcription factors, and five inhibitors was examined by PCR analyses. All PCR products were subjected to gel electrophoresis. Positive products of expected sizes for 25 Wnt and Wnt-related genes were detected (Figure 1 and Table 2). The identities of these products were confirmed through sequence analysis.

**Table 2:** Wnt components identified in human trabecular meshwork (TM) cells.

**Components in Wnt pathway**	**Genes examined**	**Genes identified**
Wnt ligand	*Wnt1*, Wnt2*, *Wnt2b*, *Wnt3*, Wnt3a*, *Wnt4*, *Wnt5a*, *Wnt5b*, *Wnt6*, *Wnt7a*, *Wnt8a*, Wnt10b*, Wnt11*	*Wnt2b*, *Wnt3*, *Wnt5a*, *Wnt5b*
Transduction protein	*Dsh1*, *Dsh2*, *Dsh3*	*Dsh1*, *Dsh2*, *Dsh3*
Receptor	*Fzd1*, *Fzd2*, *Fzd4*, *Fzd5*, *Fzd7*, *Fzd8*, *LRP5*, *LRP6*	*Fzd1*, *Fzd2*, *Fzd4*, *Fzd5*, *Fzd7*, *LRP5*, *LRP6*
β-Catenin and Degradation complex	*CTNNB1*, *GSK3β*, *APC*	*CTNNB1*, *GSK3β*, *APC*
Transcription factor	TCF1*, *TCF3*, *TCF4*, *TCF7*	*TCF3*, *TCF4*, *TCF7*
Inhibitor	*Dkk1*, *Dkk2*, *sFRP1*, *sFRP2*, *sFRP3*	*Dkk1*, *Dkk2*, *sFRP1*, *sFRP2*, *sFRP3*

RT–PCR experiments were performed to detect Wnt-related gene transcripts. Primers used for the analyses are given in Table 1. The asterisk indicates that positive PCR products were obtained using MCF-7 or HEK293 cDNAs but not with cDNA from human TM cells.

To confirm that the primer sets and the PCR conditions were optimal, MCF-7 and HEK293 cDNAs were used as positive controls. Indeed, products undetected in TM cells, such as those for genes *Wnt2*, *Wnt3a*, *Wnt10b*, and *Wnt11*, were positively identified using MCF-7 DNA as the template. *TCF1* was found expressed in HEK293 cells. Most of the genes expressed in TM cells belong to the Wnt/β-catenin pathway (Table 3). However, genes linked to the β-catenin-independent pathways were also found. A few genes were members of more than one pathway (Table 3).

**Table 3:** Categorization of Wnt components identified in human trabecular meshwork (TM) cells.

**Component in Wnt pathway**	**Wnt/β-catenin pathway**	**Wnt/planar cell polarity pathway**	**Wnt/Ca^2+^ pathway**
Wnt ligand	*Wnt3*, *Wnt2b*	*Wnt5a*, *Wnt5b*	*Wnt5a*, *Wnt5b*
Transduction protein	*Dsh2*	*Dsh1*, *Dsh2*, *Dsh3*	*Dsh3*
Receptor	*Fzd1*, *Fzd5*, *Fzd7*, *LRP5*, *LRP6*	*Fzd2*	*Fzd4*, *Fzd2*
β-Catenin and Degradation complex	*CTNNB1*, *GSK3β*, *APC*	NA	NA
Transcription factor	*TCF3*, *TCF4*, *TCF7*	NA	NA
Inhibitor	*Dkk1*, *Dkk2*, *sFRP1*, *sFRP2*, *sFRP3*	NA	NA

Wnt genes that are shown to have roles in all three Wnt signaling, canonical Wnt/β-catenin, and noncanonical Wnt/planar cell polarity and Wnt/Ca^2+^ pathways were identified in human TM cells. Some of the genes such as *Wnt5a*, *Wnt5b*, *Dsh2*, *Dsh3*, and *Fzd2* have been shown to have functional roles in more than one of the pathways.

**Figure 2:**
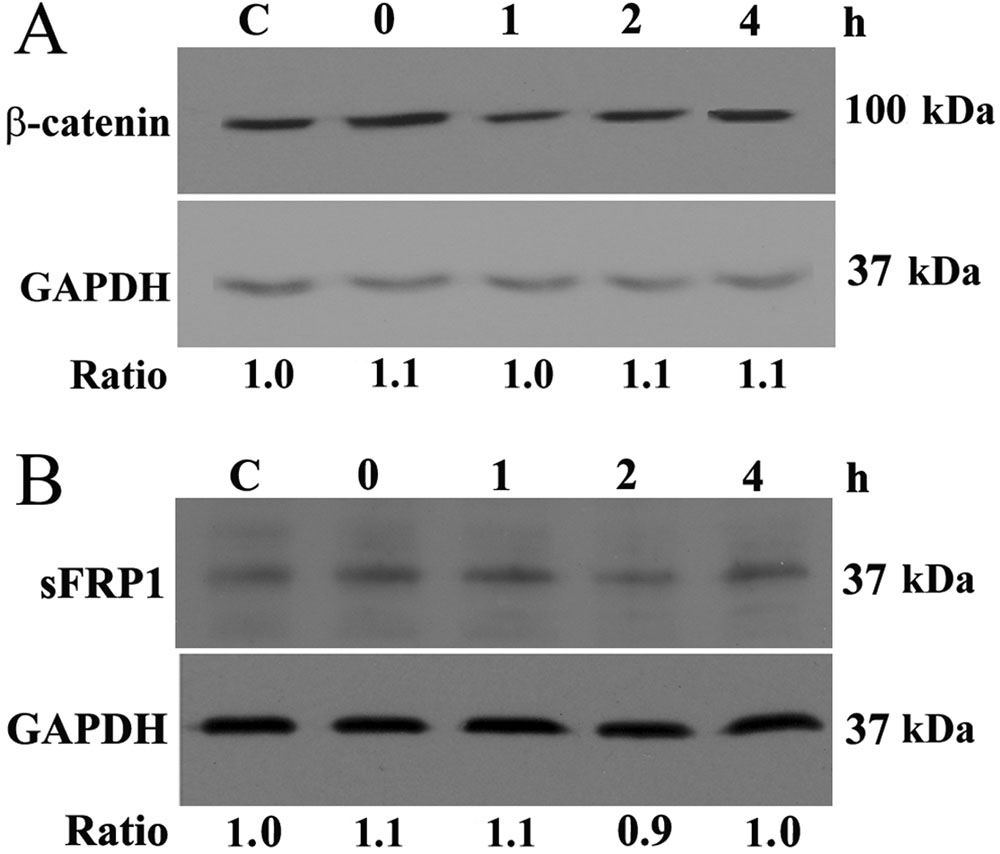
β-catenin and secreted frizzled-related protein 1 (sFRP1) protein levels in human trabecular meshwork (TM) cells. β-catenin (**A**) and sFRP1 (**B**) protein levels were assayed in human TM cells. Cells were treated with 1 mM H_2_O_2_ for 30 min. Lysates were harvested 0, 1, 2, or 4 h later. Control cells (C) were left untreated. Protein levels are expressed as ratios relative to those of glyceraldehyde 3-phosphate dehydrogenase (GAPDH). All experiments were conducted in at least three different cell lines from three different donors. Data from one representative experiment are presented.

To investigate whether changes in protein levels of Wnt-components occur after acute H_2_O_2_ treatment, western blot analyses were performed. Results indicated that there was no significant change in either β-catenin or sFRP1 protein levels (Figure 2). The ratio of β-catenin protein level relative to GAPDH for the untreated control and the 0-, 1-, 2-, and 4-h time point samples was, respectively, 1, 1.2±0.2, 1.1±0.2, 1.0±0.1, and 1.2±0.1 and that for sFRP1 was 1, 1.1±0.2, 0.9±0.2, 0.9±0.2, and 0.9±0.1.

**Figure 3:**
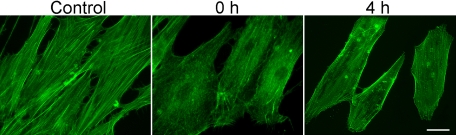
Actin staining in human trabecular meshwork cells. Cells were treated with 1 mM H_2_O_2_ for 30 min and were fixed immediately after (0 h) or 4 h later (4 h), and stained with Alexa Fluor 488-phalloidin. Cells untreated were used as controls. Results showed a significant reduction in actin stress fibers upon H_2_O_2_ treatment, a previously documented cell response [22] to oxidative stress. Scale bar represents 20 µm.

Actin staining was conducted to verify cell response to oxidative stress (Figure 3). A reduction in actin stress fibers was observed immediately following the treatment with 1 mM H_2_O_2_ for 30 min (0-h time point). The reduction persisted for at least 4 h (4-h time point).

## Discussion

In the present study we examined the expression of Wnt components in human TM cells. Wnt signaling is documented to play an important role in various biologic processes, including differentiation, development, regulation, and apoptosis of cells [31-33].

Four Wnt ligands (*Wnt2b*, *Wnt3*, *Wnt5a*, and *Wnt5b*), three Dsh genes (*Dsh1*, *Dsh2*, and *Dsh3*), seven members of the receptor complex (*Fzd1*, *Fzd2*, *Fzd4*, *Fzd5*, *Fzd7*, *LRP5*, and *LRP6*), *CTNNB1*, *GSK3β*, *APC*, three transcription factors (*TCF3*, *TCF4*, and *TCF7*), and five inhibitors (*Dkk1*, *Dkk2*, *sFRP1*, *sFRP2*, and *sFRP3*) are expressed (Figure 1 and Table 2) in human TM cells. There is a notable overlap in the Wnt and receptor genes. It is possible that the Wnt ligands bind different receptors with different affinities, resulting in different downstream effects.

Among the 25 genes we identified, most are members of the canonical β-catenin-dependent pathway (Table 3). Several others, such as *Wnt5a*, *Wnt5b*, *Dsh3*, *Fzd2*, and *Fzd4*, have been shown to be members of the noncanonical Wnt/PCP and/or Wnt/Ca^2+^pathways. Still another few have been linked to more than one pathway (Table 3). One example is Wnt5b, which is a key ligand in the Wnt/Ca^2+^ pathway and acts as an antagonist in the Wnt/β-catenin pathway in 3T3-L1 preadipocytes [34]. In addition, several Dsh and Fzd genes are common members of all three pathways (Table 3). These results suggest that all three Wnt pathways are operative in human TM cells.

The identification of *Wnt2b*, *Wnt5a*, *Fzd1*, *Fzd2*, *Fzd7*, and *sFRP1* is consistent with that reported previously by Wang and co-workers [11]. *Dkk2* and *Fzd7* were additionally found in a gene microarray study by Zhao et al. [35]. We have expanded the repertoire and indicated that, in addition to components of the Wnt/β-catenin pathway, those of the two noncanonical β-catenin-independent pathways also exist in human TM cells.

Genes, including *Wnt1* and *TCF1*, were not found in the current study. Their absence, however, is not surprising. *Wnt1* expression is observed during early development and its activity is important in determining the fate of differentiating cells [36,37]. In differentiated cells, such as TM cells, *Wnt*1 activity may be of limited significance. *TCF1* expression is mostly restricted to cells of T-cell lineage [38-40] and therefore may not be expected in TM cells.

The role of acute oxidative damage in modulating Wnt signaling in TM cells was also assessed. Two proteins, namely β-catenin and sFRP1, were chosen for western blot analysis. The former is a key element in Wnt/β-catenin signaling, and numerous studies in the literature examined the β-catenin level as a measure of Wnt activation [19,41]. The latter is of particular significance to glaucoma. As stated earlier, an increased expression of *sFRP1* has been shown to induce IOP elevation in mice [11]. Our results, however, revealed no significant changes in the levels of these two proteins upon H_2_O_2_ treatment (Figure 2). Further experiments also showed no alterations in the level of activated β-catenin (data not shown).

In the present study, the effects of oxidative stress on sFRP1 and β-catenin protein levels were evaluated for time points up to 4 h. In the literature, changes in β-catenin and sFRP1 protein levels have been shown to arise within a relatively short period of time. For instance, Aberle and co-workers [42] detected that β-catenin protein level changed 1 h after treatment with a proteosome inhibitor. Cowling et al. [43] also demonstrated that treatment with 4-hydroxytamoxifen resulted in downregulation of sFRP1 protein within 1 h. Therefore, alterations in β-catenin and sFRP1 levels, should they occur, would have been observed in our study within the time periods examined.

Previously, NIH3T3 and HEK293 cells treated with 300 μM H_2_O_2_ for 20 min were reported to yield a modest but significant activation of TCF [18]. Accumulation of β-catenin was also observed in L cells following H_2_O_2_ exposure [18]. However, in other investigations, oxidative stress was shown to negatively modulate the Wnt signal pathway [17,19]. It was documented that treatment for 24 h with 100 μM H_2_O_2_ suppressed TCF activity in murine skeletal muscle C2C12 myoblast [17], osteoblastic OB-6 [17], and HEK293 [19] cells. It seems, therefore, that the H_2_O_2_ effect may be concentration, cell-type, and/or context dependent. The 1 mM, 30 min-H_2_O_2_ treatment in our experiments had little impact on Wnt activity (Figure 2), but it did induce changes in the cytoskeletal structure in TM cells (Figure 3), as was seen in our previous experiments [22].

In conclusion, the current study revealed the existence of many Wnt elements that had not been previously tested or reported and demonstrated the presence of canonical as well as noncanonical Wnt components in human TM cells. Our results in addition indicated that the protein levels of two key Wnt signaling components, β-catenin and sFRP1, are unmodified by oxidative stress, a possible causal factor thought to be involved in glaucoma. This suggests that Wnt signaling may not be a factor related to oxidative damage in the TM system.
